# Injury Mechanisms in Mountain Biking: A Systematic Video Analysis of 534 Cases

**DOI:** 10.1002/ejsc.12327

**Published:** 2025-06-19

**Authors:** S. Bonte, C. Hartweg, A. Thouzé, E. Marcaggi, M. Dupuis, N. Graillon, P.‐J. Arnoux, L. Thollon, N. Bailly

**Affiliations:** ^1^ LBA Université Gustave Eiffel Aix Marseille University Marseille France; ^2^ Decathlon SportsLab Lille France; ^3^ Grenoble Alpes University CHU Grenoble Alpes Grenoble France; ^4^ Department of Oral and Maxillofacial Surgery APHM Conception University Hospital Marseille France; ^5^ ILab‐Spine ‐ International Laboratory on Spine Imaging and Biomechanics Marseille France

**Keywords:** crash scenario, impact conditions, injury mechanisms, mountain biking, video analysis

## Abstract

The growing popularity of mountain biking is associated with a rapid rise in injuries. Understanding the main crash scenarios is essential for improving preventive and protective measures. This study aimed to describe crash scenarios, fall kinematics, and injury mechanisms using a large video database of traumatic mountain bike crashes. A qualitative analysis was performed on 534 traumatic crash videos from a mountain biking social network. Data recorded included rider information, terrain description, cause of crashes, crash scenario, rider kinematics upon ground impact and injury. When possible, the rider's speed before the crash was measured. Among the 534 videos analysed, six specific crash scenarios were identified: forward over‐the‐bars (55.2%), forward on‐the‐bars (9.2%), sideways ejection (12.2%), sideways sliding (10.9%), collision (9.4%) and backward fall (3.2%). In the main scenario, ‘over‐the‐bars’, riders are thrown over the handlebars due to sudden deceleration, often occurring on moderate descents (53.2%) and following poor jump landings (64.4%) at speeds exceeding 30 km/h (75.3%). Tumbles were the most common impact type on the ground (64.7%), with shoulder girdle (39.7%) and upper limbs (35.6%) injuries predominating. An association between impact type on the ground and injury location was found. The mean recorded speed before the crash was 33.7 km/h, which is higher than previously reported MTB speeds. This study is the first to systematically analyse MTB accident videos, providing crucial insights to better understand full crash kinematics and help design effective protective equipment, optimize trail design, and enhance prevention campaigns on MTB slopes.

1

**Summary**

- This study is the first to systematically analyse MTB accident videos. It provides valuable information about riding speed, crash scenario and injury mechanism very useful for safety equipment manufacturer and standard committee (for helmet, back protector …), for MTB trail designer and to better tailor prevention campaigns on MTB slopes.
- Six crash scenarios were identified. Falling forward over the handlebars is the most frequent (55%) and primarily leads to shoulder girdle and upper limb injuries. It often occurs after poor jump landings at speeds exceeding 30 km/h, with the rider typically tumbling upon impact with the ground.
- The provided detailed crash description and classification are valuable to reconstruct realistic crash scenarios, and may help designing future epidemiological studies. Additionally, the developed video analysis methodology may be applied in various other sports.
- We found clear links between riding speed and the type of crash scenario, as well as between the type of injury and landing kinematics: tumbling was associated with shoulder girdle injuries, while landing flat was more often linked to upper arm injuries.

## 2 Introduction

Since the 1970s, mountain biking (MTB) has been growing in popularity. In 2015, 18.7 million MTB riders visited the European Alps (Eurac Research 2015). However, this rise in popularity has also led to an increase in MTB‐related injuries: 16.8 injuries per 1000 h of practice (Becker et al. 2013). Among these injuries, upper limb lesions dominate, accounting for 40%–75% of all injuries (Becker et al. 2013; Ehn et al. 2021; Aitken et al. 2011; Ashwell et al. 2012; Saragaglia et al. 2022; Bigdon et al. 2022). More specifically, the hands and shoulders are most prone to injury, with clavicle fractures being the most common (17.5%–62.5%) (Bigdon et al. 2022).

To continuously improve preventive measures and protective equipment, it is necessary to identify and understand the circumstances and mechanisms behind MTB injuries. While the main injury locations (Becker et al. 2013; Ehn et al. 2021; Aitken et al. 2011; Ashwell et al. 2012; Saragaglia et al. 2022; Bigdon et al. 2022) are well‐documented, and some studies describe crash scenarios by fall direction (Bush et al. 2013; Chow and Kronisch 2002), the relationship between injuries and accident circumstances remains poorly investigated. Dodwell et al. (2010) is the only study to link injuries to crash scenarios, highlighting that 78.7% of spinal injuries result from the ‘over‐the‐bars’ (OTB) scenario, where the rider is being propelled over the handlebars in a forward fall. This lack of detailed descriptions of crash scenarios limits accurate accident reconstruction and understanding of impact conditions, such as speed, force, angle… Consequently, there are no specific standards for MTB protective equipment, which is currently designed according to standards from other sports, such as cycling or motorcycling (European Committee for Standardization 2014).

Several methods have been used in safety research to obtain a detailed description of the accident: on‐the‐spot investigation (police report), interviews with the injured and, more recently, video analysis. Video analysis enable to obtain a detailed, accurate and unbiased, description of the crash kinematic and injury mechanisms, much less subjective (Steenstrup et al. 2018) than self‐reports by the injured conducted in emergency services (Saragaglia et al. 2022; Lareau and McGinnis 2011; Romanow et al. 2014). In addition, some studies have used video analysis to quantify vehicle travel speeds (Li et al. 2021) or head impact velocities (Shishov et al. 2021; Yamazaki et al. 2015). The MTB community actively shares videos online. The MTB social network pinkbike.com has a ‘Crashes’ category with more than 30,000 videos of MTB accidents uploaded by riders. Among these crashes, some are documented with injury, speed or epidemiological details, making these videos valuable for linking crash scenarios to injuries.

The goal of this study was to identify and precisely describe MTB crash scenarios and injury mechanisms through a systematic video analysis of crashes associated with reported injuries.

## 3 Methods

### 3.1 Injury Cases

The study analysed MTB crash videos published on the MTB social network ‘Pinkbike’ (Outside Inc). On this platform, users share videos and photos and comment on publications. A section of the website is dedicated to MTB crash videos, with around 2100 videos published each year. These videos are often accompanied by short comments in which the author can describe the possible injuries sustained. All videos uploaded to the website in the ‘Crashes’ section between May 2009 and May 2023 were reviewed. Inclusion criteria were:

- The crash occurred during MTB. Crashes on the street or road were excluded.
- The entire crash was recorded and visible for analysis.
- The user who posted the video reported at least one traumatic injury. A traumatic injury is defined as either a sprain, fracture, dislocation, or concussion. Other injuries such as wounds, lacerations and abrasions were excluded from the analysis.

**FIGURE 1:**
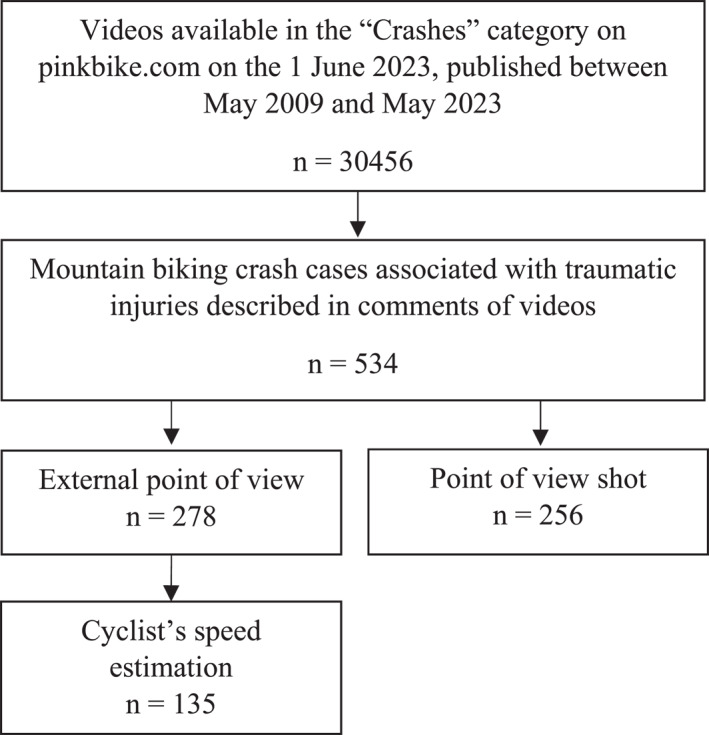
Flow chart of video acquisition and analysis processing, from the social network pinkbike.com.

Out of 30,456 videos reviewed, 534 were included in the study, with 47.8% filmed in first‐person view (point of view shot) (Figure 1).

### 3.2 Video Analysis

**FIGURE 2:**
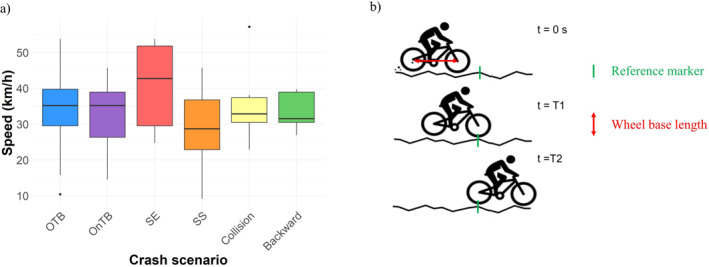
(a) Boxplot representing the link between the crash scenario (OnTB = on‐the‐bars; OTB = over‐the‐bars; SE = sideways ejection; SS = sideways sliding) and the riding speed estimate (*n* = 135 videos). (b) Illustration of the speed estimation method based on a fixed reference marker. The front (T1) and rear (T2) wheels are observed crossing a visible ground feature (green line), referred to as the reference marker. The time interval between T1 and T2 is used to compute speed as $v=\frac{{\text{WB}}_{M}}{∆T},$ where WB corresponds to the bicycle's wheelbase (red arrow).

The following information was extracted from the video:

- General information about the rider and their equipment: gender, use of helmet, use of body protection (back protector, full body armour, backpack or neck brace), type of bike (muscular or electric).
- Description of the crash scenario: causes (poor jump landing, loss of balance, excessive speed, going off track, manoeuvring or mechanical problem, collision), type of terrain (dirt ground, uneven terrain, rocks or other), slopes (steep or moderate descent, flat or ascent), crash scenario (over‐the‐bars, on the bars, sideways sliding, sideways ejection, collision, backward fall) and a description of the body‐to‐ground impact (body tumbling, flat landing; body sliding or crashing on an obstacle). The definition of the crash scenarios categories was carried out in a preliminary study on a smaller sample of MTB crash videos found on the Internet.
- The type of injury (concussion, fracture, dislocation or sprain) and the injured body part were extracted from the video description. Body parts were grouped into five anatomical regions: head, trunk (including spine, thorax, abdomen and pelvis/hip), upper extremities (arm, elbow, forearm, wrist, hand, thumb), shoulder girdle (shoulder, scapula and clavicle) and lower extremities (thigh, leg, knee, ankle, foot).
- To estimate the cyclist's speed before the crash, we measured the time taken by the bike to pass a fixed reference point on the ground (see Figure 2b). The time interval (∆*T*) and the bike's length were then used to calculate velocity with the following formula:

(1) $$V\text{elocity}=\frac{\text{Bike}\;\text{Length}}{∆T}.$$

In practice, the time interval (∆*T*) was measured from when the front wheel (T1) crossed the fixed reference point to when the rear wheel (T2) crossed it. The bike length was defined as the distance between the two wheels, known as the wheelbase (WB) (Figure 2b). A wheelbase dimension of 1.27 m was used, corresponding to a medium‐sized bike across most brands. This method was selected as it reduces velocity measurement errors associated with out‐of‐plane movements common to other measurement methods (Shishov et al. 2021). However, it introduces two potential sources of error: uncertainty in the wheelbase dimension, which typically ranges between 1.25 m (small bike) and 1.29 m (large bike) (Bikefaff 2023), and inaccuracies in measuring T1 and T2, whose precision strongly depends on the video frame rate. Consequently, an uncertainty error *ε* was calculated (Equations (2), (3), (4)) based on the video acquisition frequency (*f*) and wheelbase variations for small (1.25 m) and large (1.29 m) bikes (Bikefaff 2023).

Speed estimates with errors exceeding 30% were excluded, primarily affecting low‐frame‐rate videos. Additionally, estimates were not possible for first‐person videos, non‐linear trajectories, or when no clear reference marker was visible (due to occlusion, excessive movement or camera instability). Ultimately, speed was estimated in 135 videos (25.3%).

(2) $$v_{\text{LB}}=\frac{{\text{WB}}_{S}}{∆T+\frac{1}{2f}}\;\text{and}\;v_{\text{UB}}=\frac{{\text{WB}}_{L}}{∆T-\frac{1}{2f}},$$

with

(3) $${\text{WB}}_{S}=1.25\;m\;\text{and}\;{\text{WB}}_{L}=1.29m,$$

(4) $$\varepsilon =\frac{2\times \left(v_{\text{UB}}-v_{\text{LB}}\right)}{v}.$$

To ensure reproducibility, two investigators completed the form for 30 videos, with no observed discrepancies. For 25.3% of the videos, two experimenters independently selected the accident scheme to confirm that the categories were clearly defined and consistently applied across different evaluators.

### 3.3 Statistics

Descriptive statistics were used to describe the circumstances of the accident (user profile, terrain type, speed, etc.), the injuries sustained, and to identify the most common accident scenarios. The evaluation of how circumstances impact crash scenarios was carried out in two stages. First, Fisher's exact tests were applied to determine whether there were significant associations between different circumstance categories (such as sex, terrain type, slope, cause, speed, impact type and area injured) and the crash scenario. If a significant association was found, either Chi‐square tests or Fisher's tests (for small expected frequencies) were conducted to explore the effect of each specific level within a variable (e.g., flat slope, steep descent, moderate descent, ascent) by comparing observed and expected values. Two‐tailed *p*‐values below 0.05 were considered statistically significant. In the case of significant associations, post‐hoc analyses were performed using standardized Pearson residuals to identify cells with observed frequencies significantly different from expected frequencies. Residuals with absolute values above 1.96 were considered statistically significant (*p* < 0.05).

To assess whether rider speed influences crash scenarios or the nature of injuries, a generalized linear model (GLM) was employed with a Gaussian distribution and identity link. The variable explained was the speed when it was available (*n* = 135). The explanatory factors were ‘crash scenario’, ‘impact type’, ‘cause’ and ‘area injured’. For each categorical variable included in the model, the reference category was set as the one with the largest number of observations: OTB for crash scenario, upper limbs for area injured, rolling for impact type and bad jumps for cause of the crash. All data analysis was performed using RStudio.

### 3.4 Ethical Context

An ethical committee (CRPH no. 2024‐003) validated the study and the company Outside Inc, owner of the Pinkbike website and of the video rights, granted the right to use the video data for this study.

## 4 Results

534 crash videos with 607 reported injuries were analysed. Tables 1 and 2 show the characteristics of the mountain bikers, their crashes and their injuries.

**TABLE 1:** Characteristics of accident cases.

Crash scenario (% of injured riders)	Forward fall		Sideways fall		Collision	Backward fall	Total (% injured riders)	Fisher test *p*‐value
	Over‐the‐bars	On‐the‐bars	Sideways sliding	Sideways ejection	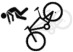	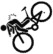		
	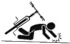	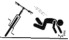	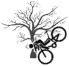	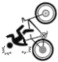				
	295 (55.2%)	49 (9.2%)	65 (12.2%)	58 (10.9%)	50 (9.4%)	17 (3.2%)	*n* = 534	
Sex (% of scenario)								0.85
Males	241 (81.7%)	34 (69.4%)	50 (76.9%)	47 (81.0%)	38 (76.0%)	14 (82.4%)	424 (79.4%)	
Females	8 (2.7%)	2 (4.1%)	2 (3.1%)	0	0	0	12 (2.2%)	
Children	3 (1.0%)	0	0	0	0	0	3 (0.6%)	
NA	43 (14.6%)	13 (26.5%)	13 (20.0%)	11 (19.0%)	12 (24.0%)	3 (17.6%)	95 (17.8%)	
Terrain type (% of scenario)**								*p* < 0.01
Dirt ground	↑ 191 (64.7%)**	21 (42.9%)	30 (46.2%)	↓ 24 (41.4%)*	22 (44.0%)	11 (64.7%)	299 (56.0%)	
Uneven ground	↓ 91 (30.8%)**	26 (53.1%)	31 (47.7%)	↑ 31 (53.4%)*	26 (52.0%)	6 (35.3%)	211 (39.5%)	
Rocks	10 (3.4%)	1 (2.0%)	3 (4.6%)	1 (1.7%)	0	0	15 (2.8%)	
Others	3 (1.0%)	1 (2.0%)	1 (1.5%)	2 (3.4%)	2 (4.0%)	0	9 (1.7%)	
Slope (% of scenario)**								
Steep descent	89 (30.2%)	↑ 22 (44.9%)*	17 (26.2%)	17 (29.3%)	12 (24.0%)	5 (29.4%)	162 (30.3%)	*p* < 0.01
Moderate descent	↑ 158 (53.6%)**	↓ 9 (18.4%)**	28 (43.1%)	22 (37.9%)	20 (40.0%)	7 (41.2%)	244 (45.7%)	
Flat	↓ 38 (12.9%)**	↑ 17 (34.7%)*	19 (29.2%)	18 (31.0%)	16 (32.0%)	3 (17.6%)	111 (20.8%)	
Ascent	10 (3.4%)	1 (2.0%)	0	1 (1.7%)	2 (4.0%)	2 (11.8%)	17 (3.2%)	
Cause (% of scenario)**								*p* < 0.001
Poor jump landing	↑ 190 (64.4%)**	34 (69.4%)	38 (58.5%)	27 (46.6%)	↓ 5 (10.0%)**	13 (76.5%)	307 (57.5%)	
Others	100 (33.9%)	14 (28.6%)	25 (38.5%)	↑ 30 (51.7%)**	↓ 10 (20.0%)*	4 (23.5%)	183 (34.3%)	
Balance loss	43 (14.6%)	6 (12.2%)	10 (15.4%)	12 (20.7%)	0	1 (5.9%)	71 (13.3%)	
Excessive speed	31 (10.5%)	1 (2.0%)	2 (3.1%)	8 (13.8%)	9 (18.0%)	0	52 (9.7%)	
Out of trail	16 (5.4%)	4 (8.2%)	4 (6.2%)	8 (13.8%)	1 (2.0%)	0	33 (6.2%)	
Manoeuvring problem	6 (2.0%)	3 (6.1%)	9 (13.8%)	2 (3.5%)	0	3 (17.6%)	23 (4.3%)	
Mechanical problem	4 (1.4%)	0	0	0	0	0	4 (0.7%)	
Collision	↓ 5 (1.7%)**	1 (2.0%)	2 (3.1%)	1 (1.7%)	↑ 35 (70.0%)**	0	44 (8.2%)	
Speed (% of scenario)								0.40
< 20 km/h	5 (1.7%)	2 (4.1%)	3 (4.6%)	0	0	0	10 (2.9%)	
20–30 km/h	14 (4.7%)	4 (8.2%)	7 (10.8%)	2 (3.5%)	1 (2.0%)	1 (5.9%)	29 (5.4%)	
30–40 km/h	43 (14.6%)	11 (22.4%)	10 (15.4%)	1 (1.7%)	4 (8.0%)	6 (35.3%)	75 (14.0%)	
> 40 km/h	15 (5.1%)	2 (4.1%)	1 (1.5%)	2 (3.5%)	1 (2.0%)	0	21 (3.9%)	
NA	218 (73.9%)	30 (61.2%)	44 (67.7%)	53 (91.4%)	44 (88.0%)	10 (58.8%)	399 (74.7%)	
Body‐to‐ground impact (% of scenario)**								*p* < 0.01
Body tumbling	↑ 191 (64.7%)**	24 (49.0%)	↓ 22 (33.8%)**	29 (50.0%)	↓ 15 (30.0%)**	↓ 4 (23.5%)*	285 (52.1%)	
Flat landing	↓ 71 (24.1%)**	18 (36.7%)	27 (41.5%)	19 (32.8%)	↑ 23 (46.0%)*	↑ 10 (58.8%)*	168 (31.5%)	
Body sliding	27 (9.2%)	7 (14.3%)	11 (16.9%)	9 (15.5%)	4 (8.0%)	3 (17.6%)	61 (11.4%)	
Body crash on an obstacle	↓ 6 (2.0%)*	0	5 (7.7%)	1 (1.7%)	↑ 8 (16.0%)**	0	20 (3.7%)	
Area injured (% of scenario)**								*p* < 0.01
Upper limbs	105 (35.6%)	21 (42.9%)	23 (35.4%)	26 (44.8%)	22 (44.0%)	3 (17.6%)	200 (37.5%)	
Shoulder girdle	117 (39.7%)	18 (36.7%)	21 (32.3%)	15 (25.9%)	13 (26.0%)	4 (23.5%)	188 (35.2%)	
Trunk	48 (16.3%)	7 (14.3%)	10 (15.4%)	10 (17.2%)	9 (18.0%)	2 (11.8%)	86 (16.1%)	
Head	50 (16.9%)	6 (12.2%)	11 (16.9%)	6 (10.3%)	5 (10.0%)	2 (11.8%)	80 (15.0%)	
Lower limbs	↓ 14 (4.7%)**	9 (18.4%)	↑ 12 (18.5%)*	12 (20.7%)	8 (16.0%)	↑ 7 (41.2%)**	53 (9.9%)	

*Note:* Expressed in percentage of injured riders. Significant differences, calculated using the Chi‐square test of independence, are indicated with * (*p* < 0.05) or ** (*p* < 0.001). ↑ indicates frequencies that are over the expected frequencies under the assumption of independence and ↓ frequencies that are under the expected frequencies.

**TABLE 2:** Area injured per detailed scenario.

Crash scenario (% of injured riders)	Forward fall					Sideways fall						Collision			Backward fall		Total, *n* = 534
	Over‐the‐bars			On‐the‐bars		Sideways sliding			Sideways ejection			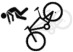			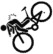		
	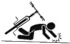			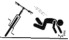		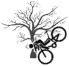			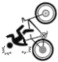								
	295 (55.2%)			49 (9.2%)		65 (12.2%)			58 (10.9%)			50 (9.4%)			17 (3.2%)		
	Col	SI	Tum	SI	Tum	Col	SI	Tum	Col	SI	Tum	Col	SI	Tum	SI	Tum	
	6 (1.1%)	99 (33.6%)	190 (35.6%)	25 (4.7%)	24 (4.5%)	5 (0.9%)	38 (7.1%)	22 (4.1%)	1 (0.2%)	28 (5.2%)	29 (5.4%)	8 (1.5%)	27 (5.1%)	15 (2.8%)	13 (2.4%)	4 (23.5%)	
Upper limbs (% of scenario)	1 (0.3%)	32 (10.8%)	72 (24.4%)	10 (20.4%)	8 (16.3%)	0	15 (23.1%)	9 (13.8%)	1 (1.7%)	11 (19.0%)	13 (22.4%)	5 (10.0%)	11 (22.0%)	7 (14.0%)	5 (29.4%)	0	200 (37.5%)
Hand/wrist	1 (0.3%)	25 (8.5%)	53 (18.0%)	7 (14.3%)	7 (14.3%)	0	11 (16.9%)	7 (10.8%)	1 (1.7%)	7 (12.1%)	7 (12.1%)	4 (8.0%)	9 (18.0%)	6 (12.0%)	4 (23.5%)	0	149 (27.9%)
Arm	0	7 (2.4%)	20 (6.8%)	4 (8.2%)	1 (2.0%)	0	5 (7.7%)	2 (3.1%)	0	4 (6.9%)	7 (12.1%)	1 (2.0%)	2 (4.0%)	1 (2.0%)	1 (5.9%)	0	55 (10.3%)
Shoulder girdle (% of scenario)	4 (1.4%)	39 (13.2%)	66 (22.4%)	12 (24.5%)	11 (22.4%)	1 (1.5%)	12 (18.5%)	4 (6.2%)	1 (1.7%)	10 (17.2%)	7 (12.1%)	0	9 (18.0%)	4 (8.0%)	5 (29.4%)	3 (17.6%)	188 (35.2%)
Trunk (% of scenario)	2 (0.7%)	12 (4.1%)	34 (11.5%)	1 (2.0%)	6 (12.2%)	1 (1.5%)	5 (7.7%)	4 (6.2%)	0	6 (10.3%)	4 (6.9%)	1 (2.0%)	6 (12.0%)	2 (4.0%)	2 (11.8%)	0	86 (16.1%)
Thorax	1 (0.3%)	7 (2.4%)	15 (5.1%)	1 (2.0%)	5 (10.2%)	1 (1.5%)	4 (6.2%)	4 (6.2%)	0	5 (8.6%)	3 (5.2%)	1 (2.0%)	3 (6.0%)	1 (2.0%)	0	0	51 (9.6%)
Spine	0	4 (1.4%)	15 (5.1%)	1 (2.0%)	1 (2.0%)	0	1 (1.5%)	0	0	1 (1.7%)	0	0	1 (2.0%)	0	1 (5.9%)	0	25 (4.7%)
Internal organs	0	2 (0.4%)	6 (2.0%)	0	1 (2.0%)	0	0	0	0	1 (1.7%)	0	1 (2.0%)	1 (2.0%)	1 (2.0%)	0	0	13 (2.4%)
Hip/pelvis	1 (0.3%)	0	4 (1.4%)	0	0	0	0	0	0	0	1 (1.7%)	0	1 (2.0%)	0	1 (5.9%)	0	8 (1.5%)
Head (% of scenario)	2 (0.7%)	22 (7.5%)	26 (8.8%)	5 (10.2%)	1 (2.0%)	1 (1.5%)	6 (9.2%)	4 (6.2%)	0	4 (6.9%)	2 (3.4%)	3 (6.0%)	2 (4.0%)	0	2 (11.8%)	0	80 (15.0%)
Concussion	2 (0.7%)	20 (3.7%)	25 (8.5%)	4 (8.2%)	0	1 (1.5%)	4 (6.2%)	1 (1.5%)	0	3 (5.2%)	2 (3.4%)	3 (6.0%)	0	0	2 (11.8%)	0	67 (12.5%)
Other area fractures	0	2 (0.7%)	1 (0.3%)	1 (2.0%)	1 (2.0%)	0	2 (3.1%)	3 (4.6%)	0	1 (1.7%)	0	0	2 (4.0%)	0	0	0	13 (2.4%)
Lower limbs (% of scenario)	0	4 (1.4%)	10 (3.4%)	1 (2.0%)	2 (4.1%)	1 (1.5%)	5 (7.7%)	6 (9.2%)	1 (1.7%)	1 (1.7%)	7 (12.1%)	0	6 (12.0%)	2 (4.0%)	6 (35.3%)	1 (11.8%)	53 (9.9%)
Ankle, foot	0	2 (0.7%)	5 (1.7%)	1 (2.0%)	1 (2.0%)	1 (1.5%)	2 (3.1%)	5 (7.7%)	1 (1.7%)	1 (1.7%)	4 (6.9%)	0	5 (10.0%)	1 (2.0%)	5 (29.4%)	1 (11.8%)	35 (6.6%)
Leg	0	2 (0.7%)	5 (1.7%)	0	1 (2.0%)	0	4 (6.2%)	1 (1.5%)	0	0	4 (6.9%)	0	1 (2.0%)	1 (2.0%)	1 (5.9%)	0	20 (3.7%)

*Note:* Col, SI and Tum corresponds to the biker body‐to‐ground impact.

Abbreviations: Col = body crash against an obstacle; SI = simple impact of the body on the ground gathering flat landing and body sliding; Tum = tumbling impact of the body on the ground.

### 4.1 Profile of the Injured and Their Injuries

Injured riders were mainly male (96.6%) (Table 1). Only four cases involved electric bikes, and seven riders were not wearing helmets. Of the 255 videos in which body protection could be seen (47.8%), full protection was noticed on 5.5% of riders, backpacks were seen on 5.1% of riders, back protectors on 3.5%, and neck braces on 3.1%. These percentages reflect only what was visible, as some protections may be concealed by larger clothing. The upper limbs (37.5%) and shoulder girdle (35.2%) were the most injured areas. Other injuries involved the trunk (16.1%)—mainly rib and sternum fractures (59.3% of trunk injuries)—head (15.0%) and lower limbs (9.9%) (Table 2). In 10% of cases, riders sustained injuries to at least two different body parts.

### 4.2 Crash Environment

Accidents primarily occurred on dirt (56.0%) or uneven terrain with roots or rocks (39.5%), mostly on moderate (45.7%) or steep descents (30.3%). The main causes, identified by the observer, were poor jump landing (57.5%), loss of balance (13.3%) and excessive speed (9.7%). Riders riding speed (*n* = 135) averaged 33.7 ± 5.1 km/h, ranging from 9.1 to 57.2 km/h, with no significant link to injury type. However, a direct relationship between the riding speed and the crash scenario was highlighted (Figure 2a). Indeed, comparatively to the ‘over‐the‐bars’ scenario, sideways ejection occurs at higher speed (Table 3; GLM Estimate = −7.03, *p* < 0.001) whereas sideways sliding happened at lower speed (Table 3; GLM Estimate = +8.91, *p* < 0.01).

**TABLE 3:** General linear model (GLM) results for speed estimation with the explanatory factors: ‘crash scenario’, ‘impact type’, ‘slope’, ‘cause’ and ‘area injured’.

Variable	Estimate	Standard error	*t*‐value	95% CI (lower)	95% CI (upper)	*p*‐value
(Intercept)	35.63	1.69	21.12	25.14	41.16	< 0.001
Schema_SS	−7.30	2.11	−3.45	−11.44	−3.16	< 0.001
Schema_SE	9.58	3.59	2.67	2.55	16.61	< 0.01
Schema_Collision	1.85	4.38	0.42	−6.73	10.43	0.67
Schema_OnTB	−2.52	2.27	−1.11	−6.96	1.92	0.28
Schema_Backward	−3.04	3.90	−0.78	−10.68	4.61	0.44
Injury_Shoulder	0.37	1.92	0.20	−3.39	4.14	0.85
Injury_Head	1.95	2.23	0.88	−2.41	6.32	0.38
Injury_Lowerlimbs	2.63	2.89	0.91	−3.04	8.30	0.37
Injury_Trunk	−2.48	2.73	−0.91	−7.83	2.87	0.37
Cause_Collision	1.29	4.05	0.31	−9.23	6.65	0.76
Cause_Others	−4.21	1.94	−2.17	−8.00	−0.41	0.03
ImpactType_Collision	−2.47	3.90	−0.64	−10.12	5.16	0.53
ImpactType_Simple_impact	−0.80	1.66	−0.48	−4.05	2.45	0.63

*Note:* Reference categories were OTB for the crash scenario, upper limbs for area injured, rolling for impact type and bad jumps for cause of the crash.

### 4.3 Detailed Crash Scenario

**FIGURE 3:**
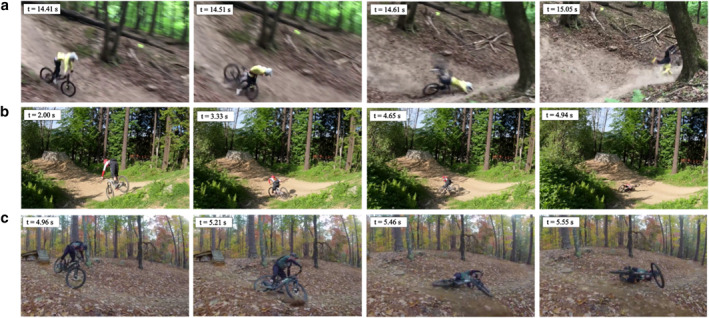
(a) Over‐the‐bars scenario, the cyclist is propelled over the handlebars and tumbles on the ground; (b) on‐the‐bars scenario, the cyclist hits the handlebars with their thorax before falling and (c) SS, the bike skids on the side with the cyclist on it, inducing falling sideways.

**FIGURE 4:**
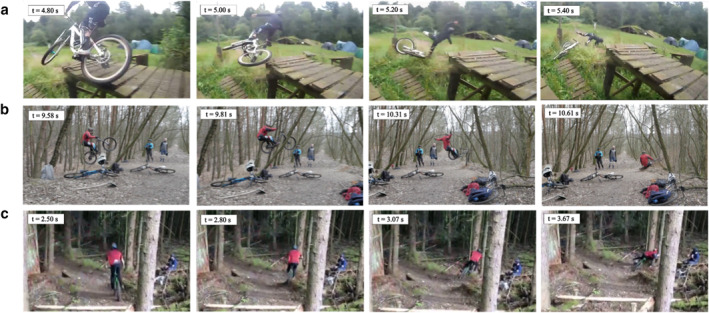
(a) Sideways ejection, the cyclist is propelled sideways out of the bike after a manoeuvre issue; (b) collision, the cyclist did not succeed to avoid a tree and collided it with their bike and (c) backward, during a jump, the cyclist released the handlebars causing a backward unbalance.

The main mechanism of injury is forward falls (64.4%), followed by sideways falls (23.1%), collisions (9.4%) and backward falls (3.2%). When falling, riders mainly tumble (52.1%) or experience a flat landing (31.5%), which affects the location of the injury: Shoulder injuries are more associated with tumbling, while upper limb injuries are mainly linked to flat landings (*p* < 0.05). Six detailed scenarios of crashes were identified:

- Forward fall—Over‐the‐bars (55.2%) (OTB) (Figure 3a): The rider is thrown over the handlebars due to sudden bike deceleration (e.g., obstacle, braking). They are more likely to tumble (64.7%, *p* < 0.01) than to land flat on the ground (24.1%, *p* < 0.001). OTB accidents mainly occur on moderate descents (53.6%, *p* < 0.001), at relatively high speeds (75.3% above 30 km/h), often after poor jump landings (64.4%, *p* < 0.001). The main injuries are to the shoulder (39.7%) and upper limbs (35.6%), while head (16.9%) and trunk (16.3%) injuries are less frequent and lower limbs injuries are rare (4.7%, *p* < 0.001).
- Forward fall—On‐the‐bars (9.2%) (OnTB) (Figure 3b): The rider falls forward onto the handlebars due to sudden deceleration, often occurring when the front wheel hits the ground after a poor jump landing (69.4%) at high speeds (68.4% above 30 km/h). Then, the cyclist typically tumbles (49.0%) or lands flat (36.7%). OnTB crashes are facilitated by steep descents (44.9%, *p* < 0.001) and flat areas (34.7%, *p* < 0.05). Injuries primarily affect the upper limbs (42.9%) and shoulder girdle (36.7%) while trunk and head injuries account for 14.3% and 12.2% of all injuries, respectively.
- Sideways—Sliding (12.2%) (SS) (Figure 3c): A sideways slide happens when the bike loses traction, causing the tires to skid beneath the rider. Riders mainly land flat on the side (41.5%) rather than tumbling (33.8%, *p* < 0.05). SS usually happen at relatively low speeds (with only 5% above 45 km/h) and are primarily due to poor jump landings (58.5%). Similar to forward falls, injuries mainly affect the upper limbs (35.4%) and shoulder girdle (32.3%), with significant injuries to the lower limbs (18.5%, *p* < 0.05). Head (16.9%) and trunk (15.4%) injuries occur in roughly one‐sixth of cases.
- Sideways fall—Ejection (10.9%) (SE) (Figure 4a): In a sideways ejection, the rider is thrown off the bike, typically due to a sudden obstacle, sharp turn, or significant loss of balance. Riders are flung off to the side and separate entirely from the bike. They are more likely to tumble (50.0%) than land flat on the side (32.8%), largely because their riding speed is generally higher than in other scenarios (Figure 4a). Poor jump landings were the main cause of SE (46.6%). However, compared to other crash scenarios, SEs were more frequently caused by other factors (51.7%, *p* < 0.05), such as excessive speed (20.7%), loss of balance (13.8%), or manoeuvring issues (13.8%). Similarly to other fall types, upper limbs (44.8%) and shoulder girdle (25.9%) injuries dominate, followed by lower limbs injuries (20.7%).
- Collision (9.4%) (Figure 4b): A collision occurs when the bike impacts a fixed or moving object, often a tree. Typically, the front wheel makes contact first, causing the bike to stop abruptly while the rider is propelled out of the bike. Depending on the angle and speed of impact, the rider may land flat on the ground (46.0%, *p* < 0.05) or crash against the obstacle (16%; *p* < 0.01). In our database, 83.3% of the collisions occurred when the cyclist was travelling at a speed above 30 km/h. Although the upper limbs were the most affected area (44.0%), there was also a high incidence of injuries to the shoulder girdle (26.0%), trunk (18.0%) and lower limbs (16.0%).
- Backward falls (3.2%) (Figure 4c): These occur when the rider tips backward, often during jumps (76.5% of poor jump landing), due to improper body positioning or failed tricks (e.g., backflip). In such falls, the cyclist tends to land directly on their back or hips. Injuries mainly affect the lower limbs (41.2%, *p* < 0.01), followed by the shoulder girdle (23.5%), head (11.8%) and upper limbs (17.6%).

## 5 Discussion

This study is the first to systematically analyse MTB accident videos, identifying crash scenarios and correlating them with injuries and crash circumstances. We identified six detailed crash scenarios with the most frequent being the forward fall over‐the‐bars, often associated with tumbles and shoulder injuries. These findings enhance our understanding of injury mechanisms in MTB and open new avenues for developing effective protective gear, optimizing trail design and enhancing prevention campaigns.

Our primary focus was on detailing crash scenarios leading to injuries, with 64.4% of cases resulting from forward falls and 23.1% from sideways falls, aligning with previous hospital studies (65%–70% vs. 25%) (Bush et al. 2013; Chow and Kronisch 2002). We refined and expanded this classification into six scenarios: over‐the‐bars, on‐the‐bars, sideways sliding, sideways ejection, backward fall and collision. The OTB scenario was previously identified as the primary crash type in MTB‐related spinal injuries (74.7%) (Dodwell et al. 2010). Our study confirms that this scenario is the most frequent in cases of spinal injury (76%) and overall (55.2%). For the first time, we also estimated the speed prior to MTB crashes. While literature suggests average MTB speeds on downhill trails of 20–25 km/h (Hurst and Atkins 2006) with peaks up to 40 km/h, our mean estimate of 33.7 km/h is higher, reflecting our focus on downhill practices and possibly indicating a higher injury risk associated with speed.

We identified the body‐to‐ground impact of the rider, noting that tumbling occurred in 52.1% of cases and flat landings in 31.5%. This distinction is critical for understanding injury mechanisms. Indeed, these impact mechanisms affect the injury location with for instance, shoulder injuries more likely to occur during tumbling. This is probably because injury mechanisms and energy dissipation differ, as tumbling can mitigate the initial impact force but may contribute to injury mechanisms like spinal hyperflexion. These fall patterns are likely influenced by the initial crash scenario and riding speed; notably, tumbling occurred more frequently in OTB incidents. Furthermore, rider reflexes may affect these mechanisms, suggesting that some impact strategies could provide greater protection than others. Further research is needed to evaluate the protective efficiency of different fall responses.

**FIGURE 5:**
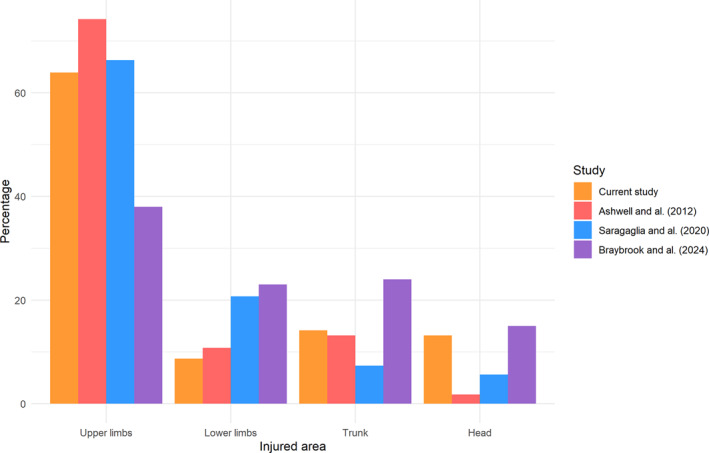
Injury distribution across four body regions: upper limbs (including here the arm, wrist, elbow and shoulder girdle), lower limbs, trunk and head, as reported in various studies. The three studies considered include Ashwell et al. (2012), which examined 898 cases of crashes which occurred in a mountain bike park; Saragaglia et al. (2022), which involved 129 patients treated at a hospital; and Braybrook et al. (2024), which focused on 329 patients. All studies were conducted in emergency departments and exclusively recorded traumatic injuries as defined in our research.

The upper limbs and shoulder girdle were the most frequently injured regions, followed by the trunk and head (Figure 5). This injury distribution, derived from a video‐based approach, mirrors patterns observed in both retrospective (Ashwell et al. 2012; Saragaglia et al. 2022) and prospective (Braybrook et al. 2024) analyses conducted in emergency departments. This consistency suggests our data is at least partially representative of global hospital trends and highlights the need to prioritize protective measures for the wrists and shoulder girdle, as well as for the trunk, which is often associated with more severe injuries (e.g., neuro problems, Dodwell et al. 2010).

The novel information on crash circumstances, scenarios, and impact kinematics can inform future developments in protective and preventive measures. We found that over half of the crashes (57.5%) were due to poor jump landings, influenced by both human and environmental factors (McNeil and McNeil 2009). Riders often exceed their skill levels on jumps, highlighting the need for campaigns to guide them towards suitable trails. Additionally, environmental factors, like improper jump design or maintenance contribute to crashes. As seen in winter sports, an engineering approach could improve jump safety by designing safer profiles based on common rider trajectories (McNeil and McNeil 2009; Petrone et al. 2017). Second, there is a lack of detailed understanding of the impact conditions during MTB crashes, such as speed, force and angle. This gap hinders the development of effective protective devices, as current MTB protective equipment is designed using standards from other sports, like cycling or motorcycling (European Committee for Standardization 2014). Our study provides a detailed description of the scenarios leading to injury and paves the way for experimental and numerical accident reconstructions that could better inform impact conditions and injury mechanisms. Our finding suggests to primarily focus on the OTB scenarios responsible for most of the shoulder and spinal injuries.

### 5.1 Limitations

Video analysis offers a repeatable, less subjective way to describe crash scenarios and kinematics than questionnaire based accident scenarios (Saragaglia et al. 2022; Lareau and McGinnis 2011; Romanow et al. 2014), but determining crash causes based on external observation may differ from the rider's perspective. Moreover, injuries were self‐reported by riders rather than being medically validated, which may affect data precision. We mitigated this by only including videos with clear injuries (e.g., fractures) and well‐described locations. Videos come from mountain bikers filming and sharing their crashes online, likely representing a specific group (young, male, risk‐takers) which may not reflect the broader MTB community. Additionally, the most serious accidents leading to death or severe injuries are unlikely to be shared online. Despite this, our crash scenarios and injury distribution closely align with previous hospital‐based studies (Ashwell et al. 2012; Saragaglia et al. 2022; Braybrook et al. 2024), suggesting some degree of representativeness. Future work using our crash scenario classification in hospital settings, or combining video analysis with medical assessments, could provide additional insights. Lastly, while this is the first study to estimate speed before MTB crashes, video quality and frame rate introduced a high error margin, up to 30%. This, combined with the need for external views, meant only 25% of videos were usable for speed estimates. Future methods, such as optical flow analysis with machine learning, could improve speed estimation, even in first‐person videos.

## 6 Conclusion

In this study, 534 MTB crashes were analysed to identify common crash scenarios and associated injuries. Forward falls, particularly ‘Over‐the‐bars’ falls, accounted for over half of all cases (55.2%), typically occurring on moderate descents, at high speeds (above 30 km/h), and due to poor jump landings. In such scenarios, riders frequently use their upper limbs to break their fall, making the shoulder girdle the most frequently injured area. These findings enhance our understanding of the relationship between fall mechanisms, impact conditions, and resulting injuries, which is crucial for developing more effective protective equipment.

## Author Contributions

All authors have made substantial contributions to all of the following: (1) the conception and design of the study, or acquisition of data, or analysis and interpretation of data, (2) drafting the article or revising it critically for important intellectual content and (3) final approval of the version to be submitted.

## Ethics Statement

The study was reviewed by the Research Committee Involving the Human Person (CRPH no. 2024‐003), at Université Gustave Eiffel, 5 Boulevard Descartes, 77,420 Champs‐sur‐Marne, France.

## Consent

The authors have nothing to report.

## Conflicts of Interest

S.B. and A.T. are employed by the company Decathlon, which manufactures bikes and protective equipment.

## Equity, Diversity and Inclusion

The author group consists of 7 men and one woman, who are junior, mid‐career and senior researchers from different medical and engineering disciplines; however, all members of the author group are from one country. Our sample is predominantly male (96.7%), consistent with prior studies (70%–92%). This also corresponds to the male‐dominant demographic on Pinkbike (94.6% in 2021) (Pinkbike 2021). It may be because men have consistently been described as more likely to take risks than women (Ruedl et al. 2010; Bailly et al. 2017).
